# Breast Cancer Subtypes in Northern Thailand and Barriers to satisfactory survival outcomes

**DOI:** 10.1186/s12885-022-10196-0

**Published:** 2022-11-08

**Authors:** Chagkrit Ditsatham, Patumrat Sripan, Benjaporn Chaiwun, Pitchayaponne Klunklin, Ekkasit Tharavichitkul, Somvilai Chakrabandhu, Pooriwat Muangwong, Imjai Chitapanarux

**Affiliations:** 1 Head Neck and Breast Unit, Department of Surgery, Faculty of Medicine, Chiang Mai University, Chiang Mai, Thailand; 2 Research Institute for Health Sciences, Chiang Mai University, Chiang Mai University, Chiang Mai, Thailand; 3 Faculty of Medicine, Chiang Mai Cancer Registry, Maharaj Nakorn Chiang Mai Hospital, Chiang Mai University, Chiang Mai, Thailand; 4 Department of Pathology, Faculty of Medicine, Chiang Mai University, Chiang Mai, Thailand; 5 Division of Radiation Oncology, Faculty of Medicine, Chiang Mai University, Chiang Mai, Thailand; 6 Northern Thai Research Group of Radiation Oncology (NTRG-RO), Faculty of Medicine, Chiang Mai University, Chiang Mai, Thailand; 7 Clinical Surgical Research Center, Faculty of Medicine, Chiang Mai University, Chiang Mai, Thailand

**Keywords:** Breast cancer, Histological subtype, Overall survival

## Abstract

**Background:**

The incidence of breast cancer (BC) in Thailand has been rising at an alarming rate. The annual incidence of BC in Thailand has doubled over a span of 15 years. A retrospective study was conducted with the primary objective of assessing and comparing survival rates of patients with BC, stratified by subtype of BC.

**Methods:**

A retrospective study was implemented for a cohort of women receiving a diagnosis of invasive BC with the objective of assessing and comparing their overall survival, stratified by BC subtype. Thai women receiving a diagnosis of their first primary invasive BC between January 2006 and December 2015 at Chiang Mai University Hospital were studied with 3,150 cases meeting the eligible criteria.

**Results:**

The median follow-up time was 4.9 years (Inter Quartile Range: 2.8–7.7). The most common diagnosed subtype was luminal B-like (n = 1,147, 36.4%). It was still the most prevalent subtype (35.8%) in women younger than 40 years and the 40–60 age-group, The proportion of patients with TNBC is the highest in women aged less than 40 years with 19.3% compared to the other age categories. Finally, among women older than 60 years, the proportion of each subtype was relatively uniform. Most women received a diagnosis of stage II disease. Triple negative subtype increased overall mortality in advanced staging (stages III and IV) (aHR:1.42, 95% CI: 0.96–2.11). The 5-year overall survival rate was found in luminal A-like at 82.8%, luminal B-like at 77.6%, HER-2 enriched at 66.4% and triple negative subtype at 64.2%.

**Conclusion:**

The histologic subtype, correlated with age and staging influenced the OS. Our results confirmed the association of triple negative BC with poor prognosis especially in advanced stage. The adjuvant medical treatment in our country could not be accessible in some group of patients, so the results of treatment and survival especially HER-2 enriched are lower than other countries without treatment barrier.

## Clinical Practice Points

Currently, breast cancer systemic treatments using endocrine treatment, chemotherapy or targeted therapy depend on the patient’s clinical status and tumor characteristics but in some countries these patients did not achieve treatment goals due to public health service policy. Thus, real-world results especially overall survival may differ from standard publication results.

## Introduction

Breast cancer (BC) is the most common female cancer and the leading cause of cancer mortality among Thai women and women worldwide. The incidence of BC in Thailand has been rising at an alarming rate. The annual incidence of BC in Thailand has doubled over a span of 15 years, from the age-standardized incidence rate (ASIR) of 17.8 per 100,000 in 1998 to 26.6 per 100,000 in 2012 [1]. The ASIR was 31.4 per 100,000 among Thai women based on data collected from 2013 to 2015 [2]. In northern Thailand, related published data showed that the ASIR of BC in a northern Thai population increased from an ASIR of 20.8 per 100,000 women-years from 1998 to 2002 to ASIR of 27.7 per 100,000 women-years from 2008 to 2012 while age-standardized mortality rates were stable around 5.0 per 100,000 women-years from 1998 to 2012 [3].

Women newly diagnosed with BC used clinical factors to determined prognosis, i.e., size and nodal status, but these did not serve as efficient predictors for the overall survival of Patients with BC because of the effectiveness of surgical and clinical interventions. Application of immunohistochemistry (IHC) in clinical settings permits subtyping BC, which can be used as another prognostic indicator [4]. Extensive studies have been conducted to estimate the overall survival of women by their BC subtype in Western countries [5–9]. Findings from these studies might be unapplicable to women from South East Asian (SEA) regions due to differences in healthcare infrastructure, personal and life-style habits or differences in genetic compositions of populations. We implemented a retrospective study of a cohort of women diagnosed with invasive BC, with the objective of assessing and comparing their overall survival, stratified by BC subtype.

## Materials and methods

### Setting

The Chiang Mai University (CMU) hospital system is a tertiary, 1,400-bed, teaching hospital serving six regions in northern Thailand. Annually, healthcare providers in the CMU hospital system provide health services to about 1,300,000 patients in outpatient and 48,000 i-inpatient departments. Since 2003, the hospital system has adopted a multidisciplinary BC treatment and management approach. The main objective has been to deliver the best healthcare services to patients with BC by providing consistent, continuous, coordinated and cost-effective care.

### Description of the cohort

Members of the cohort included Thai women receiving a diagnosis of their first primary invasive BC between January 2006 and December 2015, inclusively. The initial year of entry in the cohort was restricted to January 1, 2006 due to changes in healthcare referral policies and practices at the CMU and its network hospitals. In 2006, we implemented the institutional policy of including the HER2 biomarker as a component of the diagnostic workup of women receiving a diagnosis of any stage of invasive BC. We restricted study eligibility to women with complete pathologic diagnostic information about the status of the Estrogen Receptor (ER), Progesterone Receptor (PR) and HER2 biomarker.

The study was approved by The Medical Ethics Committee of the Faculty of Medicine, Chiang Mai University (316/2016).

### Data collection

Information on pathologic parameters (TNM staging, grade, ER, PR and HER2 status), date of surgery, considered as the date of initial diagnosis and age at the initial clinical presentation of the disease were retrieved from institutional tumor registry and validated against medical records. Additionally, information was retrieved regarding the type of surgery (lumpectomy vs. mastectomy) and adjuvant therapy by reviewing medical records. Patients’ vital status and dates of death were retrieved from databases at the Ministry of Interior, National Registration Department Vital Status.

### Definition of subtype of breast cancer

We reviewed the pathological diagnostic data and categorized BC subtypes by expression status of ER, PR and HER2.The members of the cohort were grouped by their HR and HER2 status in Luminal A-like (ER+,PR + and HER2 negative), Luminal B-like (ER+,PR- and any HER2), HER2(ER-,PR- and HER2 positive) and triple negative(ER-,PR- and HER2 negative) groups.In this study, ER and PR + defined by positive more than 1% by IHC and HER2 positive defined by IHC 2 + and confirmed with fluorescence in situ hybridization (FISH) or IHC 3+.We used “luminal-like” because our hospital did not provide ki-67 pathologic assessment Table 1).

**Table 1:** Subtyping of Breast Cancer Based on Immunohistochemistry Classification of Estrogen and Progesterone Receptors and Expression of HER2 Biomarker

Subtype	n (%)
Luminal A-like	910 (28.8)
Luminal B-like	1,147 (36.4)
HER2 Enriched	633 (20.1)
Triple-Negative	463 (14.7)
Total	3,153

### Statistical analysis

We applied descriptive statistics to summarize clinic-demographic and pathologic prognostic indictors of the members of our cohort. The variable age at initial clinical presentation of BC was categorized in (1) younger than 40 years of age; (2) 40 to 60 and (3) older than 60 years of age. The BC stages were classified as I, II, III or IV according to the American Joint Committee on Cancer (AJCC) 7th edition and overall grade as 1, 2 or 3. Chemotherapy and endocrine treatments were dichotomized as “Yes” or “No”. Women were classified by their BC subtype and differences in the distributions of clinicopathologic variables were assessed using parametric or nonparametric statistics as appropriate.

We applied Kaplan-Meier survival to assess the probability of 5-year survival by subtype of BC. For our study, we defined survival as the duration between the date of initial diagnosis and the date of death, documented in the medical records. All analyses were performed using STATA Software, Version 16 (Stata Corp, College Station, TX, USA) and hypothesis testing was two-sided with a 5% significance level.

## Results

**Fig. 1:**
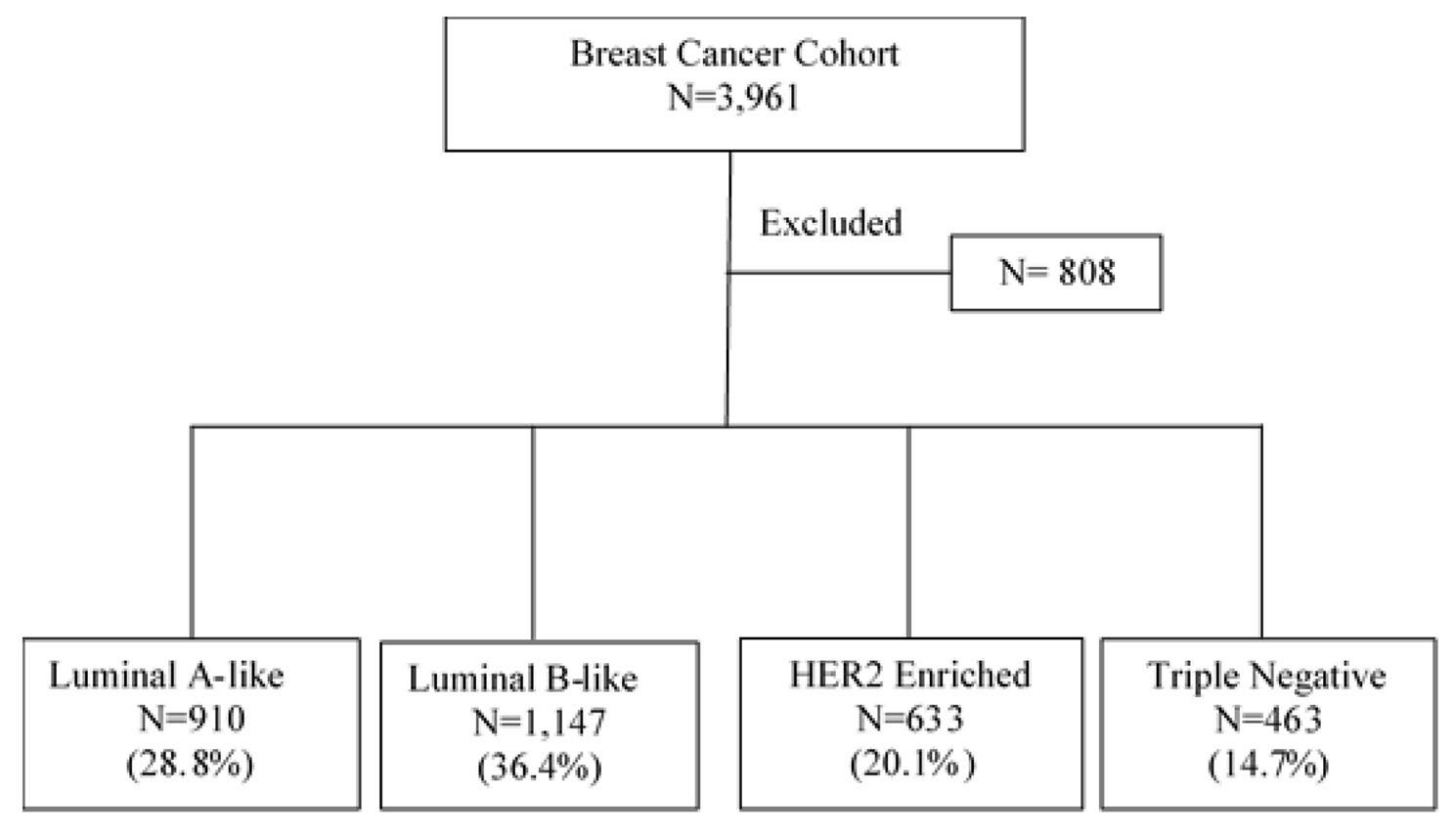
Study Flow Diagram of Breast Cancer Cohort 811 women were excluded because of incomplete pathology diagnostic information on hormone receptor (HR) status and/or HER2 biomarker

Of the 3,961 members of the cohort, 3,153 met the study eligibility criteria (Fig. 1). The median follow-up time was 4.9 years (Inter Quartile Range, IQR: 2.8–7.7). The distribution of clinicopathologic characteristics of members of the cohort at the initial clinical presentation of the disease is presented in Table 2. Overall, the most commonly diagnosed subtype was luminal B-like (n = 1,147, 36.4%) followed by luminal A-like (n = 910, 28.8%), HER2 enriched (n = 633, 20.0%) and Triple Negative (n = 463, 14.8%). However, after stratification of women by their age at the diagnosis of BC, the luminal B-like subtype was still the most prevalent in all age categories, 37.8% (131/346) among women younger than 40 years of age, 35.8% (765/2139) among the age group of 40 to 60 years and 17.81% (119/668) among women aged more than 60. The proportion of patients with TNBC is the highest in women aged less than 40 years with 19.3% (67/346) compared to the other age categories with 13.98% (299/2139) in women aged 40–60 and 14.52% (97/668) in women aged more than 60. Finally, among women older than 60 years, the proportion of women diagnosed with each subtype of BC was relatively uniform. (Table 2) Most women were diagnosed with TNM stage II, regardless of their BC subtypes; for women, diagnosed with either luminal A-like subtypes of BC, TNM stage I ranked the second highest prevalence, TNM stage I was the third most commonly diagnosed tumor stage. Information on the stage of BC was missing in the medical records of 352 women although histopathologic diagnostic data on subtype status were available and these women had completed their treatment at CMU Hospital.

**Table 2:** Clinico-pathological Characteristics of Members of the Cohort at the Initial Clinical Presentation of Breast Cancer

	Luminal A-like N (%)	Luminal B-like N (%)	HER2 Enriched N (%)	Triple-Negative N (%)	P-Value
**Age at Dx** < 40 40–60 > 60 Total	93 (10.2) 616 (67.7) 201 (22.1) 910	131 (11.4) 765 (66.7) 251 (21.9) 1,147	55 (8.7) 459 (72.5) 119 (18.8) 633	67 (14.5) 299 (64.5) 97 (21.0) 463	0.028
**TNM Staging** I II III IV Total Missing	193 (24.0) 421 (52.4) 146 (18.2) 43 (5.4) 803 107 (11.8)	201 (19.7) 457 (44.8) 274 (26.9) 87 (8.6) 1,019 128 (11.2)	77 (13.6) 247 (43.7) 172 (30.4) 69 (12.3) 565 68 (10.7)	49 (11.8) 220 (53.2) 119 (28.7) 26 (6.3) 414 49 (10.6)	< 0.001
**Tumor Grade** 1 2 3 Total Missing	58 (7.0) 572 (68.5) 205 (24.5) 835 75 (8.2)	25 (2.2) 636 (58.8) 420 (38.8) 1,081 66 (5.8)	4 (0.7) 221 (36.9) 374 (63.4) 599 34 (5.4)	8 (1.9) 201 (46.6) 222 (51.5) 431 32 (6.9)	< 0.001

Most of the patients were received chemotherapy (76.3%) and Adriamycin-based was the preferred regimen. Hormonal therapy was given to Hormone Receptor-Positive (ER + and/or PR+) patients in almost all cases (Table 3).

**Table 3:** Treatment Modalities by Breast Cancer Subtype

	Luminal A-like N (%)	Luminal B-like N (%)	HER2 Enriched N (%)	Triple-Negative N (%)	P-Value
**Surgery** No Surgery Lumpectomy Mastectomy Total	41 (4.5) 200 (22.0) 669 (73.5) 910	92 (8.0) 227 (19.8) 828 (72.2) 1,147	60 (9.5) 80 (12.6) 493 (77.9) 633	27 (5.8) 72 (15.6) 364 (78.6) 463	< 0.001
**Chemotherapy** No Yes CMF^1^ FAC/FEC/AC^2^ AC-T^3^ Total	268 (29.4) 642 (70.6) 56 (8.7) 461 (71.8) 125 (19.5) 910	307 (26.8) 840 (73.2) 30 (3.6) 610 (72.6) 200 (23.8) 1,147	100 (15.8) 533 (84.2) 20 (3.8) 371 (69.6) 142 (26.6) 633	72 (15.6) 391 (84.4) 25 (6.3) 286 (73.2) 80 (20.5) 463	< 0.001
**Radiation Therapy** No Yes Total	390 (42.9) 520 (57.1) 910	497 (43.3) 650 (56.7) 1,147	279 (44.1) 354 (55.9) 633	187 (40.4) 276 (59.6) 463	0.651
**Hormonal Therapy** No Yes Tamoxifen Aromatase Inhibitors Total	67 (7.4) 843 (92.6) 541 (76.4) 167 (23.6) 910	100 (8.7) 1,047 (91.3) 624 (72.7) 234 (27.3) 1,147	592 (93.5) 41 (6.5) 32 (78.0) 9 (22.0) 633	453 (97.8) 10 (2.2) 10 (100.0) 0 (0.0) 463	< 0.001
**Anti-HER2** No Yes Total	908 (99.8) 2 (0.2) 910	1,053 (91.8) 94 (8.2) 1,147	507 (80.1) 126 (19.9) 633	459 (99.1) 4 (0.9) 463	< 0.001

**Fig. 2:**
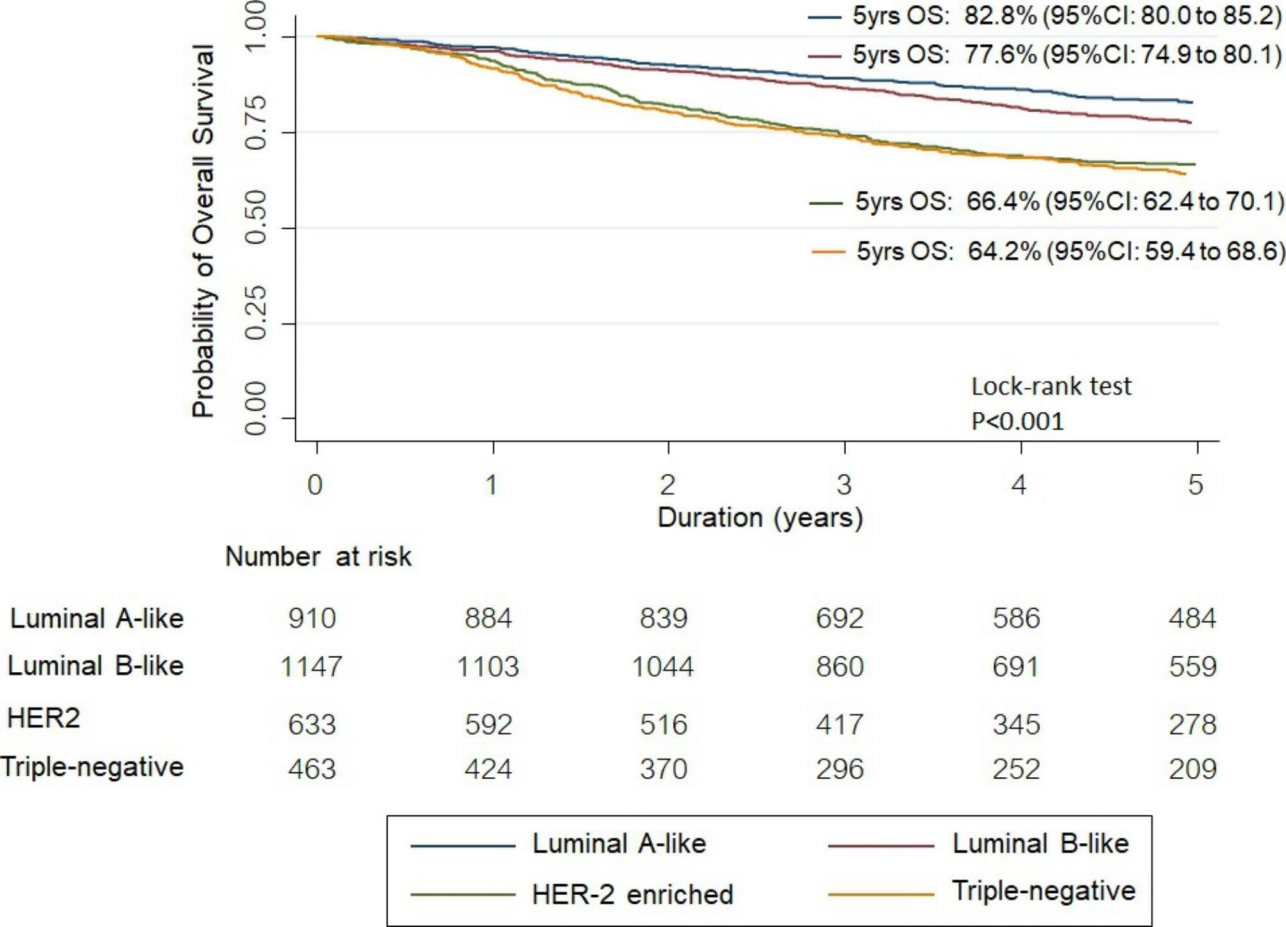
Kaplan–Meier Curve Overall Survival in Each Classification of Breast Cancer Subtypes

The triple-negative subtype increased overall mortality in advanced staging (stage III and IV) and (aHR:1.42, 95% CI: 0.96–2.11,p-value = 0.083) by multivariable cox regression analysis however tumor subtype did not affect the mortality in early-stage BC (stage I-II) (Table 4). The best 5-year OS rate was found in luminal A-like (82.8%), followed by luminal B-like (77.6%). HER-2 enriched and triple-negative subtype had an inferior survival rate. The lowest 5-year OS rate was found in the triple-negative subtype (64.2%) as shown in Fig. 2.

**Table 4:** Risk of Overall Mortality by Subtype of Breast Cancer, Stratified by Stage

	Hazard Ratio (95% Confidence Interval)			
	**Stages I and II**	**p-value**	**Stages III and IV**	**p-value**
**Luminal A-like**	1		1	
**Triple Negative**	0.65 (0.38–1.13)	0.130	1.42 (0.96–2.11)	0.083
**Luminal B-like**	0.81 (0.59–1.11)	0.186	1.21 (0.92–1.59)	0.173
**HER2 Enriched**	0.75 (0.44–1.28)	0.290	1.01 (0.70–1.48)	0.944

*Risk was estimated by multivariable Cox proportional hazard regression adjusted for age at diagnosis, surgical, chemo, and radiation therapies. The Luminal A-like is the reference

## Discussion

Our study demonstrated the influences of subtype at diagnosis on the survival of Northern Thai BC patients. Most of our patients were luminal B-like. The second most common type was luminal A-like which also had the highest survival. Patients with triple-negative and HER-2 enriched had an increased adjust hazard risk of death compared to luminal-like subtypes. This result is consistent with other studies [5–7]. However, the triple negative subtype patients in this cohort did not increase the risk of death compared to luminal-like and HER2 enriched subtype that may be from most of the patients was found in the early-stage group. Chemotherapy was also given for HR+, HER2-subtypes patients in our study because of the other prognostic factors ex. histologic grade, presence of lymphovascular invasion, and nodal status were included for considering adjuvant treatment. Some hormonal receptor positive (HR+) Patients with BC in our study did not receive hormonal therapy and a few triple negative Patients with BC received hormonal therapy could not identified the reason from medical record. Those cases similar to ATLAS trial that enrolled women with HR- or unknow in study [10].

Most Thai patients were covered under the medical welfare scheme which can access public health services from public hospitals and private hospitals registered with The National Health Security Office (NHSO). This scheme of government universal coverage offers the three cornerstones of BC treatment: surgery, chemotherapy, and radiotherapy. However, NHSO restricts access to certain critical medical treatments such as novel chemotherapy or anti-hormonal therapy, and limited anti-HER2 treatment only in the adjuvant setting in nodal positive disease. National Comprehensive Cancer Network (NCCN) and some studies recommended adjuvant taxane-containing chemotherapy in patients who had axillary lymph node-positive, trastuzumab in HER2 positive [8–11] but the taxane-containing regimen has been approved for adjuvant treatment in node-positive BC and metastatic BC (MBC) since 2007, adjuvant trastuzumab was just approved in 2015 and eligible for BC patients who had node positive only in universal health coverage (UHC),Social security scheme(SSS) and Civil Servant Medical Benefit Scheme. and inflammatory BC and supraclavicular lymph node metastasis was not allowed to use adjuvant trastuzumab and Patients with MBC also could not access trastuzumab.

In this study cohort, HER-2 enriched group survival was 66.4% and shorter than the related published OS between 75 and 85% [5, 7, 11] because our patient was enrolled before the approval of adjuvant trastuzumab treatment which may have been the reason for an inferior outcome but similar results were found with other studies from Thailand [12, 13]. In our study, the result of luminal A-like 5-year OS was 83.4% in all stages which was less than in other studies [14–16]. Nonsteroidal aromatase inhibitor was approved to use in 2009. NSHO did not allow steroidal aromatase inhibitors, fulvestrant and CDK4/6 inhibitors for HR + and anti-HER2 for patients with metastatic BC. The further evaluation of treatment efficacy should compare between two periods of treatment “before and after” accessing adjuvant treatment.

## Conclusion

Histological subtype correlated with age and staging affected to OS. Our results confirmed the association of triple-negative BC with poor prognosis especially in high grade tumors and advanced stage. The adjuvant medical treatment in our country could not be accessed by some groups of patients, so the results of treatment and survival especially HER-2 enriched appear inferior to those of other countries without treatment barrier.

## Data Availability

The datasets used or analysed during the current study available from the corresponding author on reasonable request.

## Abbreviations

BC: Breast cancer
ASIR: Age-standardized incidence rate
HR: Hormonal receptor
ER: Estrogen receptor
PR: Progesterone receptor
OS: Overall survival
